# Emodin Attenuates Rheumatoid Arthritis by Modulating the NF-κB/HIF-1α/VEGF Signaling Pathway

**DOI:** 10.3390/ijms27083460

**Published:** 2026-04-12

**Authors:** Dehao Du, Yihang Lou, Linlan Zhou, Jiayu Tian, Tingdan Zhang, Zexuan Qiu, Xiaofeng Rong

**Affiliations:** 1 Department of Combination of Chinese and Western Medicine, The First Affiliated Hospital of Chongqing Medical University, Chongqing 400016, China; dudehao715@163.com (D.D.); zhoulinlan1235pm@163.com (L.Z.); jiayutian0116@163.com (J.T.); 15223413265@163.com (T.Z.); 13067038559@163.com (Z.Q.); 2 Institute of Literature in Chinese Medicine, Nanjing University of Chinese Medicine, Nanjing 210023, China; yihanglou@icloud.com

**Keywords:** rheumatoid arthritis, emodin, NF-κB, hypoxia, pannus

## Abstract

This study aims to evaluate the therapeutic efficacy of emodin (EMO) in rheumatoid arthritis (RA) and to verify whether its underlying mechanism involves the blockade of pathological angiogenesis via the inhibition of the nuclear factor-kappa B (NF-κB)/hypoxia-inducible factor-1α (HIF-1α)/vascular endothelial growth factor (VEGF) signaling axis. Bovine type II collagen-induced arthritis (CIA) mouse models and lipopolysaccharide (LPS)-stimulated EA.hy926 endothelial cells were utilized in this study. The effects of EMO on joint pathological alterations, the expression of NF-κB/HIF-1α/VEGF axis proteins, inflammatory cytokines (tumor necrosis factor-alpha (TNF-α), interleukin-6 (IL-6), and interleukin-1 beta (IL-1β)), and angiogenic capacity were assessed using histopathological analysis, Western blotting, immunohistochemistry (IHC), immunofluorescence, and tube formation assays. Furthermore, small interfering RNA (siRNA) interference targeting key molecules was employed to validate the molecular mechanisms underlying the therapeutic effects of EMO. In the CIA model group, the ankle joints of mice exhibited pronounced inflammatory infiltration, synovial hyperplasia, and bone destruction. Compared with the model group, both the EMO and methotrexate (MTX) treatment groups demonstrated attenuated synovial hyperplasia and cartilage destruction, along with significantly downregulated expression levels of key NF-κB pathway proteins, HIF-1α, and VEGF in joint tissues (*p* < 0.001). In vitro experiments revealed that EMO treatment significantly reduced the LPS-induced secretion of pro-inflammatory cytokines (TNF-α, IL-6, and IL-1β) (*p* < 0.001), and decreased both the number and total length of tubular structures formed by endothelial cells compared to the control (*p* < 0.001). Notably, siRNA-mediated knockdown of *p65* resulted in decreased intracellular protein levels of HIF-1α and VEGF, accompanied by a significant reduction in tube formation (*p* < 0.001). This study demonstrates that EMO alleviates pathological damage in RA by inhibiting the activation of the NF-κB signaling pathway, which subsequently downregulates pathological angiogenesis and inflammatory responses mediated by the HIF-1α/VEGF axis. These findings provide a robust experimental basis for the potential application of EMO as a therapeutic agent for RA.

## 1. Introduction

Rheumatoid arthritis (RA) is a prevalent autoimmune disease pathologically characterized primarily by synovitis, pannus formation, and subsequent cartilage and bone destruction [1]. Affecting approximately 1% of the global population, its etiology is significantly influenced by genetic factors, age, and adverse lifestyle habits. Without timely and effective therapeutic intervention, the disease progressively worsens, leading to severe damage to cartilage and bone tissues and, ultimately, disability [2].

Inflammatory responses and abnormal angiogenesis mutually promote each other and collectively drive the pathogenesis of RA [3]. The transcription factor NF-κB plays a central role in inflammatory regulation [4]; its activation (e.g., the upregulation of P65 and IKK-β) induces the release of multiple pro-inflammatory cytokines (such as TNF-α, IL-6, and IL-1β), thereby exacerbating the intra-articular inflammatory microenvironment [5,6]. Concurrently, inflammatory infiltration and the abnormal proliferation of synovial tissue elevate oxygen consumption [7,8]. The resulting hypoxic state in the synovium induces the expression of hypoxia-inducible factor-1α (HIF-1α), which in turn upregulates vascular endothelial growth factor (VEGF) and angiopoietin-1 (ANG-1), consequently promoting pathological angiogenesis [3,9,10]. Notably, the NF-κB and HIF-1α signaling pathways are frequently synergistically activated during the pathological progression of RA [11,12], forming a positive feedback loop between inflammation and angiogenesis. Therefore, identifying targets or pharmacological agents capable of simultaneously inhibiting both pathways is of substantial clinical value for RA treatment.

Although conventional therapeutics, including methotrexate (MTX), glucocorticoids, and non-steroidal anti-inflammatory drugs (NSAIDs), are widely applied in clinical practice [13,14,15], their utility is often constrained by limited efficacy and adverse side effects. Hence, there is an urgent need to explore novel, effective therapeutic agents and targets to better control disease progression. Traditional Chinese medicine (TCM) monomers, characterized by their multi-target effects and relatively low toxicity, have emerged as a promising avenue for RA treatment research [16,17,18]. Emodin (EMO; molecular formula: C15H10O5), a natural anthraquinone derivative, exhibits a broad spectrum of pharmacological activities [19]. Our research group has a long-standing interest in the pathogenic mechanisms of RA and the pharmacological effects of EMO. While our previous studies have demonstrated the robust anti-inflammatory and bone-protective properties of EMO [20,21,22,23], its specific impact on pannus formation remains completely unexplored. Furthermore, it remains unclear whether EMO can simultaneously intervene in synovial inflammation and pathological angiogenesis by modulating the NF-κB/HIF-1α/VEGF signaling axis to alleviate RA, and the precise molecular mechanisms involved have yet to be elucidated.

This study aims to systematically evaluate the therapeutic efficacy and underlying molecular mechanisms of EMO in the treatment of RA. We hypothesize that EMO impedes inflammation-driven angiogenesis by inhibiting the activation of the NF-κB pathway and downregulating the expression of HIF-1α and VEGF, ultimately alleviating synovitis, suppressing pannus formation, and mitigating joint destruction.

## 2. Results

### 2.1. Histopathological Changes in the Ankle Joints of CIA Mice

**Figure 1:**
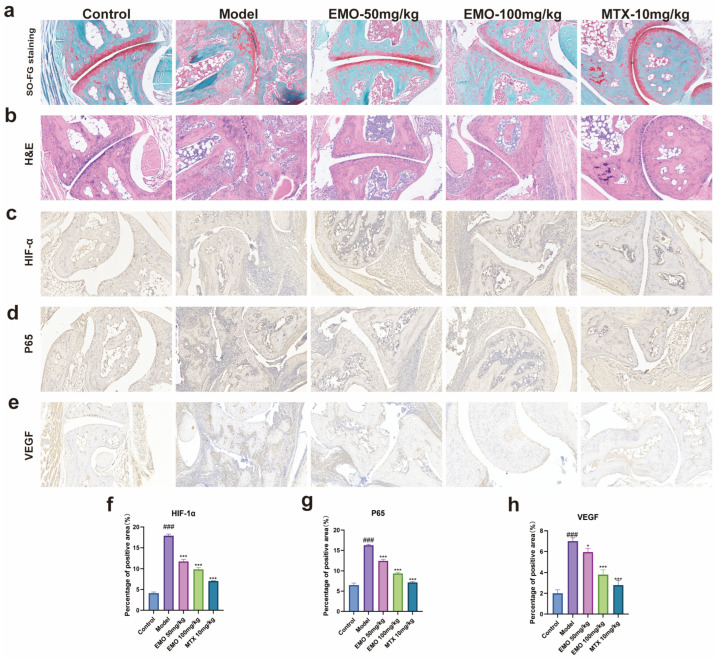
Histological effects of EMO on collagen-induced arthritis (CIA) mice. (**a**) Safranin O/Fast Green staining indicates profound cartilage matrix loss and chondrocyte disorganization in the model group, which was notably restored in the EMO groups (200×, n = 8/group). (**b**) H&E staining shows intact structures in the control group, whereas the model group displays synovial hyperplasia, inflammatory cell infiltration, and bone destruction. EMO/MTX treatments alleviated these pathological changes (200×, n = 8/group). (**c**–**h**) Representative IHC images and quantitative analysis of mouse ankle joint tissues (200×, n = 8/group). ### *p* < 0.001 vs. the normal group; * *p* < 0.05, *** *p* < 0.001 vs. the model group. Data were analyzed using one-way ANOVA followed by Tukey’s multiple comparison test and are presented as the mean ± SD.

Hematoxylin and eosin (H&E) staining revealed that the ankle joints of mice in the control group possessed intact structural integrity and regularly arranged synovial layers without obvious abnormalities (Figure 1a). Conversely, the model group exhibited significantly narrowed joint cavities, extensive synovial fibroblast hyperplasia, massive inflammatory cell infiltration into the sub-synovial layer, and localized bone erosion. In contrast, mice treated with EMO or MTX exhibited negligible inflammatory infiltration, cellular proliferation, or bone erosion (Figure 1b).

Safranin O-Fast Green staining demonstrated that the articular cartilage in the control group possessed a smooth surface, orderly aligned chondrocytes, and uniform red matrix staining (Figure 1c). In the model group, the cartilage surface was rough and irregular, with a severe loss of the red-stained matrix, accompanied by a markedly reduced and disorganized chondrocyte population. The EMO and MTX treatment groups maintained relatively intact cartilage morphology, preserved matrix staining intensity, and displayed a more regular arrangement of chondrocytes.

### 2.2. Expression of NF-κB Pathway-Related Proteins in CIA Mice

**Figure 2:**
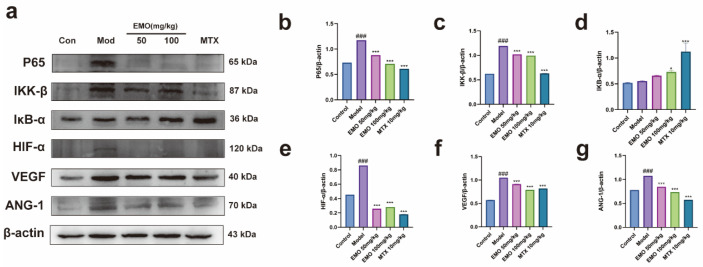
Effects of EMO on the expression of NF-κB and HIF-1α pathway-related proteins in the periarticular tissues of CIA mice. (**a**) Western blot analysis of p65, IKK-β, IκB-α, HIF-1α, VEGF, and ANG-1 protein expression, with β-actin serving as the internal control. (**b**–**g**) Quantitative densitometry analysis of the corresponding bands. ### *p* < 0.001 vs. the normal group; * *p* < 0.05, *** *p* < 0.001 vs. the model group (n = 3). Data were analyzed using one-way ANOVA with Tukey’s multiple comparison test and are presented as the mean ± SD.

The expression levels of NF-κB pathway-related proteins in the paw synovium and periarticular soft tissues of CIA mice were evaluated using Western blotting (Figure 2). Compared with the control group, the protein expression levels of p65 and IKK-β were significantly elevated in the model group (*p* < 0.001), while IκB-α expression was markedly diminished (*p* < 0.001). Following treatment with EMO and MTX, the expression levels of p65 and IKK-β were significantly downregulated compared to the model group (*p* < 0.001), concurrently with a restoration of IκB-α expression (*p* < 0.05, *p* < 0.001). Inter-group comparisons among the EMO and MTX groups revealed significant differences in p65 and IKK-β expression across all groups (*p* < 0.001). For IκB-α expression, no significant difference was observed between the EMO 100 mg/kg group and the EMO 50 mg/kg group (*p* > 0.05), whereas significant differences existed among the other groups (*p* < 0.001).

**Figure 3:**
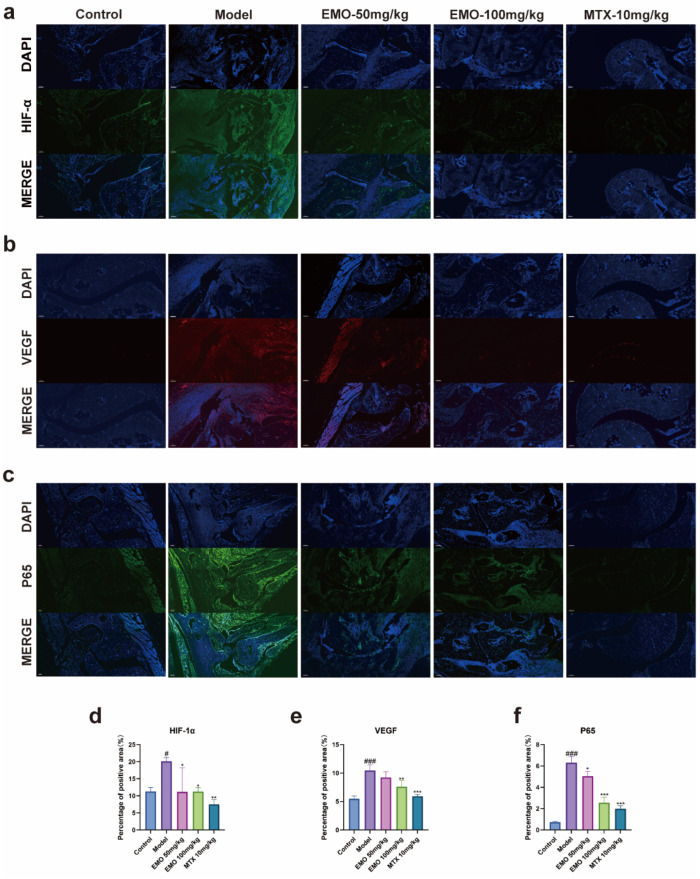
Immunofluorescence staining showing the expression and localization of angiogenesis- and inflammation-related proteins in the periarticular tissues of CIA mice. (**a**,**b**) Representative images illustrating the expression and distribution of HIF-1α (**a**) and VEGF (**b**) across the control, model, and EMO/MTX treatment groups. (**c**) IF staining of p65. Nuclei were counterstained with DAPI (blue), and target proteins are indicated by specific fluorescence (HIF-1α and p65 in green, VEGF in red). Images were acquired using a laser confocal microscope at 200× magnification. (**d**–**f**) Quantitative analysis of the corresponding images (positive area %). # *p* < 0.05, ### *p* < 0.001 vs. the normal group; * *p* < 0.05, ** *p* < 0.01, *** *p* < 0.001 vs. the model group. Data are presented as the mean ± SD.

These findings were further corroborated by IHC and immunofluorescence (IF) staining. Compared to the normal group, joint tissue sections from the model group exhibited strong positive expression of p65 (*p* < 0.001). Conversely, the positive staining area and intensity of p65 were significantly attenuated in the EMO and MTX groups (*p* < 0.001) (Figure 1d and Figure 3c).

### 2.3. Expression of Pannus-Related Proteins in CIA Mice

Western blotting was employed to detect the expression of angiogenesis-related proteins (Figure 2). Compared to the control group, the protein levels of HIF-1α, VEGF, and Ang-1 were significantly upregulated in the model group (*p* < 0.001). Treatment with EMO or MTX significantly decreased the expression of these proteins (*p* < 0.001). Group comparisons revealed significant differences in HIF-1α and Ang-1 expression among the EMO and MTX groups (*p* < 0.001). Notably, VEGF expression was significantly lower in the EMO 100 mg/kg group compared to the EMO 50 mg/kg group (*p* < 0.01), whereas the MTX 10 mg/kg group exhibited a comparatively modest reduction (*p* < 0.05).

IHC and IF analyses further validated the high expression and altered spatial distribution of HIF-1α and VEGF in the model group tissues (*p* < 0.001), which were characterized by reduced staining intensity and smaller positive areas in the EMO and MTX groups (*p* < 0.001) (Figure 1c,e and Figure 3a,b).

### 2.4. Expression of the NF-κB Pathway and Inflammatory Cytokines In Vitro

**Figure 4:**
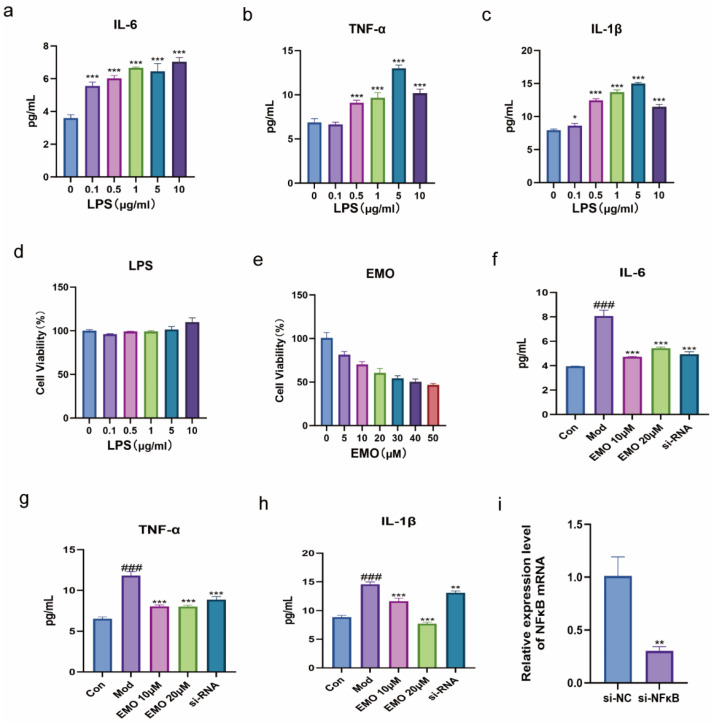
CCK-8, qPCR, and ELISA assays evaluating the effects of EMO on LPS-induced cellular inflammation and siRNA efficiency. (**a**–**c**) ELISA quantification of secreted pro-inflammatory cytokines (IL-6, IL-1β, and TNF-α) in the culture supernatants to determine the optimal LPS concentration. (**d**,**e**) CCK-8 assay assessing cell viability under various EMO concentrations to establish non-cytotoxic treatment doses. (**f**–**h**) ELISA quantification of secreted IL-6, IL-1β, and TNF-α. (**i**) qPCR analysis of siRNA knockdown efficiency and *p65* mRNA levels; *GAPDH* served as the internal reference. ### *p* < 0.001 vs. the normal group; * *p* < 0.05, ** *p* < 0.01, *** *p* < 0.001 vs. the model group (n = 3). Data are presented as the mean ± SD.

Based on preliminary CCK-8 and ELISA screening, the optimal inflammatory stimulation concentration of LPS was determined to be 1 μg/mL. EMO treatment concentrations were set at 10 μM and 20 μM, and the siRNA concentration was optimized to 100 nM based on qPCR knockdown efficiency (Figure 4a–e,i) (Table 1).

**Figure 5:**
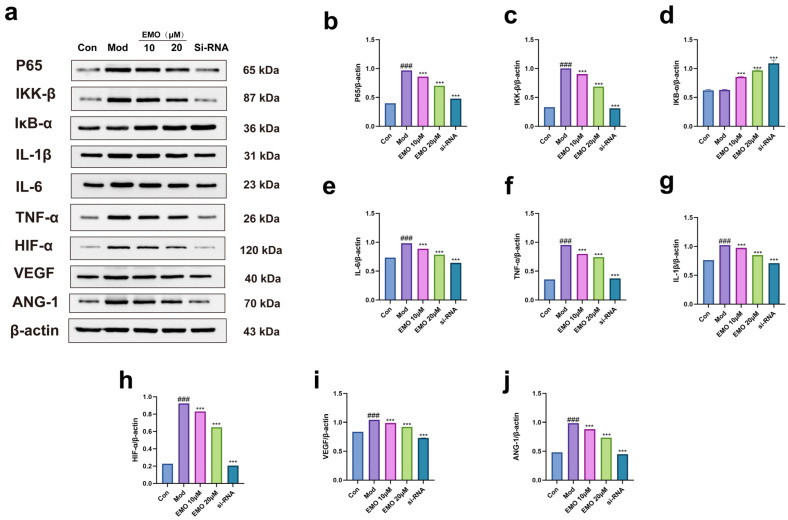
EMO inhibits LPS-induced activation of the NF-κB and HIF-1α pathways. Following preliminary screening via CCK-8 and ELISA assays, cellular inflammation was induced using 1 μg/mL LPS. The treatment concentrations for EMO were established at 10 μM and 20 μM, and the siRNA concentration was set at 100 nM based on qPCR results. (**a**) Representative Western blot images detecting the expression of NF-κB pathway-related proteins (p65, IKK-β, IκB-α), inflammatory cytokines (IL-6, IL-1β, TNF-α), and angiogenesis-related proteins (HIF-1α, VEGF, Ang-1), with β-actin serving as the internal control. (**b**–**j**) Quantitative densitometry analysis of the corresponding bands. ### *p* < 0.001 vs. the normal group; *** *p* < 0.001 vs. the model group (n = 3). Data were analyzed using one-way ANOVA followed by Tukey’s multiple comparison test and are presented as the mean ± SD.

Western blot and ELISA assessments of the NF-κB pathway and pro-inflammatory cytokines (Figure 4f–h and Figure 5a–g) revealed that LPS stimulation significantly upregulated the protein levels of p65 and IKK-β (*p* < 0.001), accompanied by a substantial surge in the secretion of IL-6, IL-1β, and TNF-α (*p* < 0.001). Conversely, IκB-α protein levels were significantly diminished (*p* < 0.001). EMO treatment effectively suppressed the LPS-induced elevations of p65, IKK-β, and the aforementioned cytokines (*p* < 0.001), while restoring IκB-α expression (*p* < 0.001). Significant inter-group differences were observed for all these markers among the various treatment groups (*p* < 0.001).

**Figure 6:**
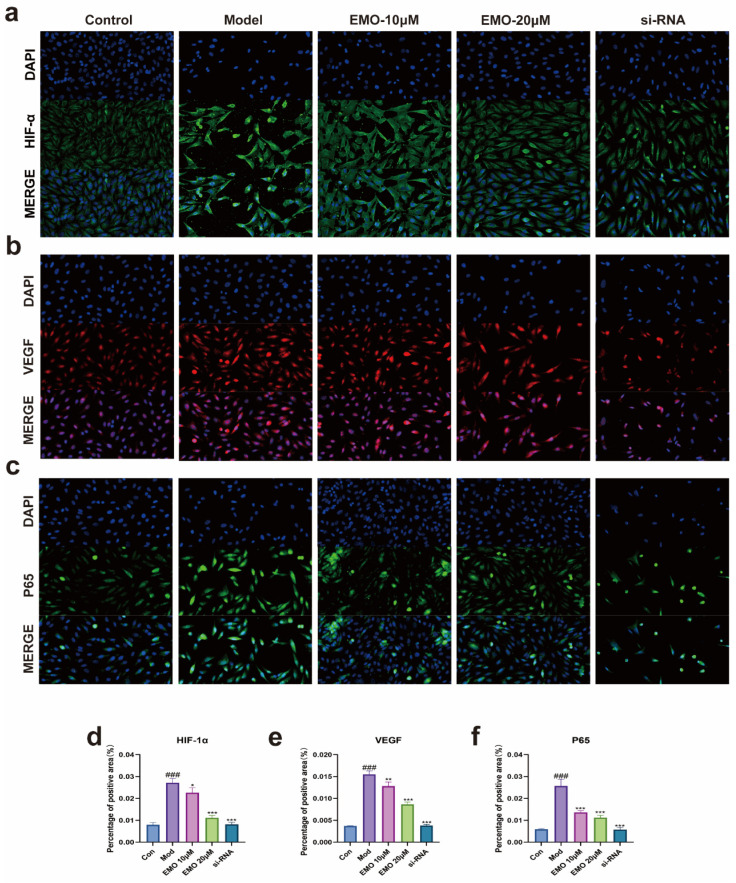
Immunofluorescence staining of angiogenesis- and inflammation-related proteins in cells. (**a**,**b**) Representative images of HIF-1α (**a**) and VEGF (**b**) expression and distribution. (**c**) IF staining of p65. Target proteins are shown in green (HIF-1α, p65) and red (VEGF). Images were captured using a fluorescence microscope at 200× magnification. (**d**–**f**) Quantitative analysis of positive area (%). ### *p* < 0.001 vs. the normal group; * *p* < 0.05, ** *p* < 0.01, *** *p* < 0.001 vs. the model group. Data are presented as the mean ± SD.

IF staining further confirmed this regulatory mechanism: LPS-stimulated cells displayed an intense nuclear fluorescent signal for p65, indicating predominantly nuclear localization (Figure 6c). In contrast, EMO treatment significantly weakened the nuclear p65 signal (*p* < 0.001), with the fluorescence predominantly retained in the cytoplasmic region.

### 2.5. Effects of EMO on Angiogenesis-Related Protein Expression In Vitro

Western blot analysis of pro-angiogenic proteins (Figure 5a,h–j) demonstrated that HIF-1α, VEGF, and Ang-1 levels were significantly elevated in the model cells compared to the control (*p* < 0.001). EMO treatment significantly mitigated the expression of these proteins (*p* < 0.001). Significant differences were noted in the expression of HIF-1α, VEGF, and Ang-1 among the different treatment groups (*p* < 0.001).

**Figure 7:**
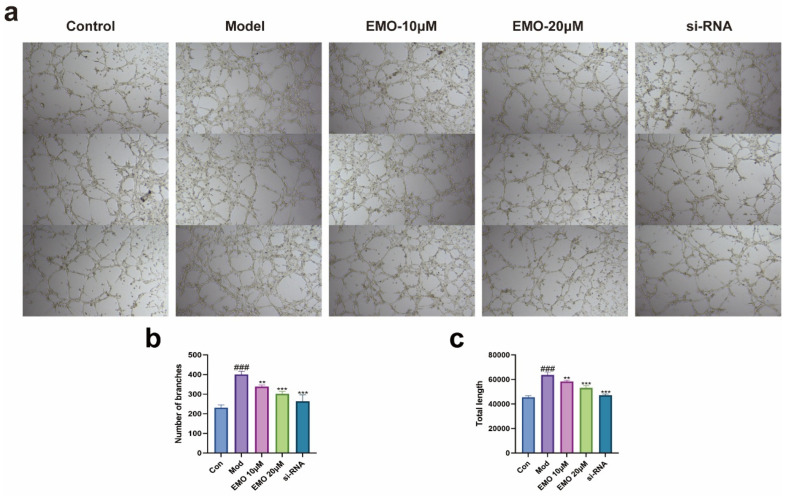
Tube formation assay evaluating the impact of EMO on LPS-induced angiogenesis. (**a**) Representative micrographs (Magnification: 100×): the control group shows minimal network formation; the model group (LPS 1 μg/mL) exhibits significantly enhanced network formation, which is inhibited by EMO (10, 20 μM). (**b**,**c**) Quantitative analysis of the capillary networks, including total tube length and number of meshes. ### *p* < 0.001 vs. the normal group; ** *p* < 0.01, *** *p* < 0.001 vs. the model group. Data are presented as the mean ± SD.

These changes were corroborated by IF analysis (Figure 6a,b): model cells exhibited high-intensity fluorescent signals for HIF-1α and VEGF, with a high proportion of positive cells (*p* < 0.001). EMO treatment significantly weakened these signals and reduced the number of positive cells (*p* < 0.01, *p* < 0.001). Furthermore, the in vitro tube formation assay aligned with the molecular findings: endothelial cells in the model group formed a significantly increased number of closed tubular structures (*p* < 0.001). Conversely, EMO treatment suppressed tube formation, resulting in a network density markedly lower than that of the model group (*p* < 0.01, *p* < 0.001) (Figure 7).

## 3. Discussion

Rheumatoid arthritis (RA) is an autoimmune disease primarily characterized by chronic synovitis [24]. The pathogenesis initiates with the aberrant activation of synovial and immune cells [25], followed by a sustained inflammatory cascade, synovial hyperplasia, and destructive erosion, ultimately culminating in cartilage degradation and bone destruction [26,27,28]. Inflammatory cells residing within the synovium secrete an abundance of pro-inflammatory cytokines (such as TNF-α, IL-1β, and IL-6), which not only directly damage joint architecture but also recruit and activate osteoclasts, thereby accelerating bone resorption [29,30]. Furthermore, localized metabolic alterations and the hypoxic microenvironment exacerbate the maintenance and expansion of this inflammatory network [7,31]. This pathological progression is the consequence of intricate interactions among multiple cell types (synovial fibroblasts, endothelial cells, macrophages, and lymphocytes) and signaling pathways, dictating that effective therapeutic strategies must simultaneously target both inflammation and structural deterioration [31,32].

NF-κB represents a crucial family of transcription factors responsible for regulating a myriad of inflammatory and immune response genes [4]. Its canonical activation involves the phosphorylation and subsequent degradation of IκB, which liberates the p65 complex to translocate into the nucleus and initiate the transcription of downstream inflammatory genes.

In the pathology of RA, NF-κB drives the production of pro-inflammatory cytokines, promotes synovial fibroblast proliferation and the secretion of matrix-degrading enzymes, and supports osteoclast differentiation, thereby directly participating in the perpetuation of joint inflammation and bone destruction [5,6]. Consequently, inhibiting the NF-κB signaling pathway can concurrently attenuate pro-inflammatory cytokine levels, mitigate synovial hyperplasia, and suppress osteoclastic activity, making it a highly compelling therapeutic target for RA. In the present study, the model group exhibited significant upregulation of p65 and IKK-β, alongside the downregulation of IκB-α, indicating robust NF-κB activation. Treatment with EMO effectively restored IκB-α levels, suppressed p65 expression, and reduced inflammatory cytokine secretion, substantiating its potent inhibitory effect on this pathway.

Pannus is a pathological tissue characterized by abnormal angiogenesis, frequently observed in chronic inflammatory or autoimmune conditions [33,34]. Composed of newly formed capillaries, inflammatory cells, and a fibrous matrix, pannus aggressively invades articular cartilage and bone, leading to localized tissue destruction and functional impairment [35]. In RA, inflammatory stimuli trigger synovial hyperplasia accompanied by extensive neovascularization; these nascent vessels, together with the proliferating fibrous tissue, constitute the pannus that spreads across the cartilage or bone surface [36].

Localized hypoxia, induced by inflammatory infiltration and tissue proliferation, is a pivotal driver of pannus formation. Hypoxic or inflammatory signals stabilize HIF-1α, which subsequently upregulates downstream angiogenic factors (such as VEGF and Ang-1). This cascade promotes endothelial cell proliferation, migration, and tube formation, establishing an aberrant microvascular network [3,37]. Inhibiting pathological angiogenesis not only restricts the delivery of nutrients and inflammatory cells to the lesion but also disrupts the positive feedback loop between inflammation and angiogenesis. Thus, it holds profound therapeutic significance for alleviating synovial hyperplasia, restraining erosive lesions, and preserving joint architecture. Our IHC and IF results revealed elevated and widely distributed expression of HIF-1α and VEGF in the model group, which aligned with the enhanced capillary network observed in the tube formation assay, collectively supporting the promotive role of pathological angiogenesis in this model.

Emodin (EMO), a natural anthraquinone derivative, is renowned for its diverse biological activities, including significant anti-inflammatory, antioxidant, and immunomodulatory effects. Previous studies have highlighted its formidable therapeutic potential in various inflammatory disease models [38,39,40]. Our research group has a long-standing commitment to elucidating the pathological mechanisms of RA and the pharmacological properties of EMO. Our prior investigations into Compound Dahuang Powder have firmly established its anti-inflammatory and bone-protective efficacies in RA [41,42]. As the sovereign herb (principal component) in this traditional formulation, Rhubarb’s core active ingredient, EMO, has been previously validated by our team for its therapeutic effects on CIA mice and inflammatory cells [20,21,22,23]. Consistent with these pharmacological characteristics, the in vivo results of the current study confirm the profound reversing effect of EMO on RA pathogenesis. Histopathological analysis demonstrated that EMO treatment not only significantly ameliorated synovial hyperplasia and inflammatory infiltration but, crucially, effectively arrested cartilage matrix degradation and bone erosion. These morphological improvements strongly correlate with the observed reduction in histopathological joint damage, compellingly indicating that EMO exerts substantial protection over damaged joint structures by comprehensively ameliorating the articular microenvironment.

To further delineate the underlying mechanisms, we evaluated the alterations in relevant signaling molecules. The in vitro results showed that EMO treatment significantly decreased the expression levels of pro-inflammatory cytokines (IL-6, IL-1β, TNF-α) and pro-angiogenic factors (HIF-1α, VEGF, Ang-1). Concurrently, at the signal transduction level, the expression of key signaling proteins p65 and IKK-β was downregulated, whereas the level of the inhibitory protein IκB-α was elevated, and the nuclear translocation of p65 was markedly restrained. These molecular data suggest that the anti-inflammatory and bone-protective mechanisms of EMO are likely intimately associated with its specific inhibition of the NF-κB signaling pathway, thereby circumventing the downstream “inflammation-angiogenesis” cascade mediated by the HIF-1α/VEGF axis.

Notably, this study also unveils the unique advantage of EMO in modulating pathological angiogenesis. Our data confirm that while downregulating the expression of HIF-1α, VEGF, and Ang-1, EMO significantly suppressed the tube-forming capacity of endothelial cells. Furthermore, recent studies have explicitly confirmed that EMO can significantly downregulate CD31 expression and exert potent anti-angiogenic effects in other pathological models [43]. Given that NF-κB is a critical transcriptional regulator of HIF-1α, we hypothesized and verified that EMO likely exerts its effects via the “NF-κB/HIF-1α/VEGF” axis. Specifically, by inhibiting NF-κB activity, EMO indirectly downregulates the transcription of HIF-1α and its downstream target VEGF, thereby restricting neovascularization within the pannus and severing the supply lines that deliver nutrients and inflammatory cells to the inflamed tissues.

Furthermore, in vitro mechanistic validation experiments demonstrated that specific knockdown of p65 expression using siRNA partially replicated the effects of EMO in inhibiting inflammatory cytokine secretion and attenuating endothelial cell tube formation in the cellular model. This finding further substantiates that NF-κB is positioned critically upstream of EMO’s action and is an indispensable link for EMO to exert its inhibitory function on the pro-inflammatory/pro-angiogenic axis. Taken together, the findings of this study robustly support the conclusion that EMO manifests its therapeutic potential in RA by specifically targeting the NF-κB/HIF-1α/VEGF pathway.

It is worth emphasizing, however, that the precise modality by which EMO acts on HIF-1α remains incompletely elucidated. Therefore, subsequent experiments, including chromatin immunoprecipitation (ChIP), reporter gene assays, and proteasome inhibition tests, are warranted to verify the regulation of p65 on the *HIF1A*/*VEGFA* promoters and to determine whether EMO influences the degradation pathway of HIF-1α. Furthermore, given its well-documented multi-target characteristics, EMO might also modulate HIF-1α activity through alternative, non-oxygen-dependent pathways, such as the AKT/mTOR cascade, potentially bypassing NF-κB entirely [44].

Beyond these molecular mechanisms, several macroscopic and pharmacological limitations of the present study should be acknowledged. First, although the downregulation of VEGF and HIF-1α indirectly reflects the suppression of angiogenesis, direct visual and quantitative evaluations of pannus vascularization—such as CD31 immunohistochemical staining or in vivo Doppler angiography—were not performed. Second, dynamic clinical parameters reflecting the functional status of the joints, including continuous joint swelling measurements, daily 4-point clinical scoring, and weight distribution tests, were not comprehensively documented throughout the disease progression. Finally, the precise pharmacokinetic profile of EMO, particularly its specific accumulation in the synovial fluid, remains to be determined, making it challenging to establish an exact in vivo and in vitro dose equivalence. Future investigations integrating robust pharmacokinetic tracking, dynamic functional scoring, and direct angiographic imaging are necessary to fully map the comprehensive therapeutic profile and clinical translatability of Emodin in rheumatoid arthritis.

## 4. Materials and Methods

### 4.1. Materials and Reagents

Emodin (EMO; molecular weight 270.24 g/mol, molecular formula C15H10O5, purity 99.20%) was purchased from MedChemExpress (MCE, Monmouth Junction, NJ, USA). Methotrexate (MTX; 2.5 mg/tablet) was obtained from Xin Yiping (First Affiliated Hospital of Chongqing Medical University, Chongqing, China).

### 4.2. Establishment of the CIA Mouse Model

Forty male DBA/1 mice (8 weeks old, weighing 18–20 g) were purchased from Chengdu Yaokang Biotechnology Co., Ltd. (Chengdu, China). All animal experiments were approved by the Animal Ethics Committee of Chongqing Medical University (Approval No.: 2022-K296). The mice were housed in a specific pathogen-free (SPF) environment maintained at 25 ± 2 °C with a 12 h light/12 h dark cycle and provided ad libitum access to food and water. The randomization was based on body weight and initial clinical presentation to ensure baseline comparability.

On day 1, equal volumes of Complete Freund’s Adjuvant (CFA; Cat. 190288, Chondrex, Woodinville, WA, USA) and Bovine type II collagen (CII; Cat. 20021, Chondrex, USA) were thoroughly emulsified on ice. Excluding 8 mice in the normal control group, the remaining mice were subcutaneously injected with 100 µL (2 mg/mL) of the emulsion at the base of the tail. On day 21, a booster immunization was administered using an equal volume of a mixture containing Incomplete Freund’s Adjuvant (IFA; Cat. 210332, Chondrex, USA) and CII. From day 22 onwards, the immunized mice were randomly divided into four groups (n = 8 per group) based on body weight and initial clinical presentation to ensure baseline comparability: the model group (administered oral saline), the EMO 50 mg/kg/day group, the EMO 100 mg/kg/day group, and the MTX group (10 mg/kg/week). MTX was administered once weekly based on standard clinical and murine experimental protocols due to its specific pharmacokinetic and toxicity profile, while Emodin was administered daily. The selected doses (50 and 100 mg/kg/day) were based on our group’s previous studies [22,45]. Treatments were administered via daily oral gavage for 21 consecutive days. The entire experiment lasted for a total of 42 days, and on day 43, the mice were euthanized via an intraperitoneal (i.p.) injection of sodium pentobarbital (150 mg/kg; Merck KGaA, Darmstadt, Germany).

### 4.3. Histopathological Analysis

Following euthanasia, the ankle joints of the mice were harvested and fixed in 4% paraformaldehyde for 24 h. The tissues were decalcified using EDTA at room temperature, embedded in paraffin, and sectioned at a thickness of 4 µm. The sections were subsequently subjected to Hematoxylin and Eosin (H&E) and Safranin O-Fast Green staining. Histological changes were observed and photographed using an optical microscope (Leica, Wetzlar, Germany).

### 4.4. Immunohistochemistry (IHC)

Paraffin sections were dewaxed, rehydrated, and subjected to microwave-assisted antigen retrieval in a citrate buffer. After blocking for 10 min, the sections were incubated overnight at 4 °C with primary antibodies against HIF-1α (20960-1-AP, Proteintech, Wuhan, China, 1:200), p65 (80979-1-RR, Proteintech, 1:200), and VEGF (81323-2-RR, Proteintech, 1:200). On the following day, after washing at room temperature, the sections were incubated with a goat anti-rabbit secondary antibody (SA00004-2, Proteintech, 1:200) for 50 min. Visualization was performed using 3,3′-diaminobenzidine (DAB), followed by hematoxylin counterstaining, dehydration, clearing, and mounting. Images were captured using a Leica microscope. IHC images were quantitatively analyzed using ImageJ software (version 1.53).

### 4.5. Cell Culture

EA.hy926 cells were purchased from Wuhan Procell Life Science and Technology Co., Ltd. (Wuhan, China). The cells were cultured in DMEM supplemented with 10% fetal bovine serum (FBS) and 1% penicillin-streptomycin, and maintained in a humidified incubator at 37 °C with 5% CO_2_.

### 4.6. Establishment of Cell Model Concentrations

(1) EA.hy926 cells were seeded in 96-well plates at a density of 4 × 10^3^ cells/well. Upon adhesion, cells were stimulated with 0.1, 0.5, 1, 5, and 10 µg/mL of lipopolysaccharide (LPS; MCE, USA) for 24 h. The supernatants were collected to measure the levels of inflammatory cytokines (TNF-α, IL-6, and IL-1β) using ELISA kits (Jiubang Bio, Jiujiang, China) to determine the optimal modeling concentration.
(2) Under the same conditions, cells were treated with 5, 10, 20, 30, 40, and 50 µM EMO and incubated for 24 h. Subsequently, 10 µL of CCK-8 reagent (Beyotime, Shanghai, China) was added to each well and incubated at 37 °C for 1 h. The optical density (OD) was measured at 450 nm to calculate cell viability, identifying the non-cytotoxic concentrations (10 and 20 µM) for subsequent experiments.

### 4.7. siRNA Transfection and Quantitative Real-Time PCR (RT-qPCR)

Complexes were prepared by mixing 100 µM of siRNA targeting *p65* with Lipofectamine™ 3000 (Invitrogen, Carlsbad, CA, USA) according to the manufacturer’s instructions. The mixture was added dropwise to the cells in antibiotic-free medium and incubated at 37 °C for 8 h. The medium was then replaced with complete medium for a further 72 h of culture. Total RNA was extracted using TRIzol reagent (Beyotime, Shanghai, China), and reverse transcription was performed using the PrimeScript™ RT kit (TaKaRa, Kusatsu, Japan). mRNA expression levels were quantified via SYBR^®^ Green qPCR (TaKaRa). Primers were synthesized by Sangon Biotech. The amplification conditions were as follows: 95 °C for 10 min, followed by 40 cycles of 95 °C for 15 s and 60 °C for 60 s. *GAPDH* served as the internal reference, and relative expression was calculated using the 2^−ΔΔCt^ method.

### 4.8. Enzyme-Linked Immunosorbent Assay (ELISA)

Based on the conditions determined in Section 4.6, cells were divided into the following groups: normal group, model group (1 µg/mL LPS), low-dose EMO group (1 µg/mL LPS + 10 µM EMO), high-dose EMO group (1 µg/mL LPS + 20 µM EMO), and siRNA group (1 µg/mL LPS + siRNA). Supernatants were collected, and the levels of TNF-α, IL-6, and IL-1β were measured according to the ELISA kit instructions (Jiubang Bio).

### 4.9. Western Blotting

Total protein was extracted from EA.hy926 cells and CIA mouse paw tissues using RIPA lysis buffer supplemented with 1% PMSF. Following protein quantification via the BCA assay (Beyotime), 5× loading buffer was added, and the samples were denatured at 95 °C for 10 min. Proteins were separated by SDS-PAGE and transferred onto PVDF membranes. The membranes were blocked with 5% non-fat milk for 2 h at room temperature and then incubated overnight at 4 °C with primary antibodies against p65, IKK-β, IκB-α, HIF-1α, VEGF, and ANG-1 (all at 1:1000, Proteintech). After washing with TBST, the membranes were incubated with HRP-conjugated secondary antibodies (1:5000) for 1.5 h at room temperature. Protein bands were visualized using an ECL detection system (BIO-RAD, Hercules, CA, USA) and quantitatively analyzed using ImageJ software.

### 4.10. Immunofluorescence (IF)

Cells were seeded in 6-well plates and treated with LPS and EMO according to their respective groups for 24 h. Concurrently, paraffin-embedded mouse joint tissues were dewaxed. Samples were fixed with 4% paraformaldehyde for 15 min, permeabilized with 0.3% Triton X-100 for 10 min, and blocked with 5% BSA for 30 min. The samples were then incubated overnight at 4 °C with primary antibodies against HIF-1α, p65, and VEGF (all at 1:200). After incubation with fluorescently labeled secondary antibodies (1:200) for 30 min at room temperature, the nuclei were counterstained with DAPI. Images were acquired using a Nikon laser confocal microscope (Nikon, Tokyo, Japan).

### 4.11. Endothelial Cell Tube Formation Assay

Matrigel (Beyotime) was thawed and uniformly coated in a 48-well plate on ice, then allowed to polymerize in a 37 °C incubator. EA.hy926 cells were seeded at a density of 1 × 10^4^ cells/well and treated with the conditioned media from the respective groups (as described in Section 4.6). Tube formation was photographed under a microscope, and the number of meshes and total tube length were quantified using ImageJ software.

### 4.12. Statistical Analysis

Statistical analyses were performed using GraphPad Prism 10 software (GraphPad Software, San Diego, CA, USA). The Shapiro–Wilk test was initially used to assess data normality. Data conforming to a normal distribution were analyzed using one-way analysis of variance (ANOVA) followed by Tukey’s post hoc test for multiple comparisons; non-normally distributed data were analyzed using appropriate non-parametric tests. Results are expressed as the mean ± standard deviation (SD), and a *p*-value < 0.05 was considered statistically significant.

## 5. Conclusions

In conclusion, this study provides compelling evidence from both animal and cellular models that EMO can effectively inhibit NF-κB signaling, thereby downregulating HIF-1α/VEGF-associated pro-angiogenic and pro-inflammatory responses, ultimately alleviating synovitis and joint tissue destruction. These findings strongly support the strategy of integrating inflammation inhibition with anti-angiogenesis for the treatment of RA. Future endeavors should focus on more intricate mechanistic validations and clinically translatable evaluations to further confirm the feasibility and safety window of EMO as a promising candidate therapeutic molecule.

## Figures and Tables

**Table 1:** PCR primer sequence.

Gene	Primer	Sequence (5′–3′)	PCR Products
*GAPDH*	Forward Reverse	GGGGCTCTCCAGAACATC TGACACGTTGGCAGTGG	173
*P65*	Forward Reverse	GCGAATGGCTCGTCTGT TCGCACTTGTAGCGGAAG	184

## Data Availability

The original contributions presented in this study are included in the article. Further inquiries can be directed to the corresponding author.

## Abbreviations

The following abbreviations are used in this manuscript:

EMO	Emodin
RA	Rheumatoid arthritis
CIA	Collagen-induced arthritis
LPS	Lipopolysaccharide
TNF-α	Tumor necrosis factor-alpha
IL-6	Interleukin-6
IL-1β	Interleukin-1 beta
MTX	Methotrexate
NF-κB	Nuclear factor-kappa B
HIF-1α	Hypoxia-inducible factor-1α
VEGF	Vascular endothelial growth factor
ANG-1	Angiopoietin-1
NSAIDs	Non-steroidal anti-inflammatory drugs
TCM	Traditional Chinese medicine
